# Quality and Flavor Difference in Dry-Cured Meat Treated with Low-Sodium Salts: An Emphasis on Magnesium

**DOI:** 10.3390/molecules29102194

**Published:** 2024-05-08

**Authors:** Jun Xiang, Xuejiao Wang, Chaofan Guo, Liping Zang, Houde He, Xiaoyu Yin, Jianping Wei, Jianxin Cao

**Affiliations:** 1 Faculty of Food Science and Engineering, Kunming University of Science and Technology, Kunming 650500, China; xiangjun19990903@163.com (J.X.); guochaofanfan@outlook.com (C.G.); zangliping1999@163.com (L.Z.); mocha588@outlook.com (H.H.); yinxiaoyu1234@163.com (X.Y.); 2 College of Food Science and Technology, Northwest University, Xi’an 710000, China; jianpingwei0327@nwu.edu.cn

**Keywords:** dry-cured meat, low-sodium salt, taste, aroma, magnesium ion, salt reduction

## Abstract

The present study aimed to develop low-sodium curing agents for dry-cured meat products. Four low-sodium formulations (SPMA, SPM, SP, and SM) were used for dry-curing meat. The physicochemical properties and flavor of the dry-cured meat were investigated. The presence of Mg^2+^ ions hindered the penetration of Na^+^ into the meat. The weight loss, moisture content, and pH of all low-sodium salt groups were lower than those of S. Mg^2+^ addition increased the water activity (*A*_w_) of SPMA, SPM, and SM. Dry-curing meat with low-sodium salts promoted the production of volatile flavor compounds, with Mg^2+^ playing a more prominent role. Furthermore, low-sodium salts also promoted protein degradation and increased the content of free amino acids in dry-cured meat, especially in SM. Principal component analysis (PCA) showed that the low-sodium salts containing Mg^2+^ were conducive to improving the quality of dry-cured meat products. Therefore, low-sodium salts enriched with Mg^2+^ become a desirable low-sodium curing agent for achieving salt reduction in dry-cured meat products.

## 1. Introduction

Dry-meat products crafted from whole pieces, such as dry-cured loin, ham, bacon, and jerked beef, are highly valued and enjoyed globally for their distinct sensory qualities and extended shelf life [1]. Generally, a significant amount of salt is added to dry-cured meat products to reduce moisture and extend preservation time. For example, the salt content in dry-cured ham in Europe varies from 4.5% to 8% [2], while bacon ranges from 1.65% to 3.7% in Australia [3]. However, in China, the amount of salt added to dry-cured meat far exceeds that of other countries, especially in ham, where it can reach as high as 7% to 12% [4]. Excessive consumption of sodium salts increases the risk of hypertension, cardiovascular disease, stroke, liver and kidney disease, gastric cancer, and osteoporosis, etc. [5]. The World Health Organization (WHO) stated that the daily intake of salt for adults should be less than 5 g (2 g/sodium). Therefore, sodium reduction in meat products, especially dry-cured meat products, has been garnering increasing attention.

Currently, various physical processing methods are used to reduce sodium addition in dry-cured meat products, such as high pressure, ultrasonic methods, and electric field treatment [6]. There are also other methods, such as directly reducing the amount of NaCl [7], mediated pickling for salt reduction [8], and the utilization of low-sodium salts [9]. Among these methods, the partial replacement of NaCl with other metal salts is the most widely used method in traditional dry-cured meat.

Previous studies have shown that reducing sodium content can affect the quality of meat products, including sensory characteristics, flavors, bacterial colonies, and texture. Armenteros et al. [10] and Wu et al. [11] demonstrated that using 50% KCl as a replacement for NaCl in hams resulted in a more pronounced bitter taste. Wu et al. [12] found that replacing over 40% of sodium with potassium significantly altered the distribution of volatile compounds in dry-cured meat, leading to changes in aroma and flavor. Research by Lee et al. [13] indicated that in the dry-cured ham, replacing over 50% of NaCl with KCl led to a significant decrease in tenderness, while cooking loss increased with higher concentrations of KCl. Nachtigall et al. [14] revealed that in the salted meat, under the same ionic strength, calcium chloride (CaCl_2_) exhibited higher oxidative capacity and had a more pronounced impact on lipid profiles compared to NaCl and KCl. Li et al. [15] demonstrated that in dry-cured bacon, 40% KCl effectively inhibited the formation of biogenic amines, body alkalinity, and histamine while increasing the residual nitrate level. These findings indicated that different metal ions and substitution ratios have varying impacts on the quality of dry-cured meat products. Therefore, it is essential to explore suitable substitution levels and formulas to avoid affecting the quality of dry-cured meat.

Taking into consideration the high sodium level in dry-cured meat and the impacts linked with salt reduction on the quality and sensory properties, this study used four formulations of low-sodium salt during the processing of dry-cured meat. By measuring the physicochemical parameters, aroma, and taste of dry-cured meat products, we aim to select composite salts to enhance the quality of dry-cured meat while achieving the highest reduction in sodium content. This research will provide a theoretical basis and technical support for the development of low-sodium dry-cured meat products.

## 2. Results

### 2.1. Effect of Low-Sodium Salts on the Physicochemical Properties of Dry-Cured Meat 

#### 2.1.1. Metal Ion Content, Weight Loss, Moisture, Water Activity, and Water State of Dry-Cured Meat

Table 1 provides the measured content, theoretical content value, and permeability of each metal ion in dry-cured meat products. The contents of Na^+^, K^+^, and Mg^2+^ in each group of samples were proportional to the formula content, which was consistent with expectations. The amount of Na^+^ added was relatively low in the groups SPMA, SPM, and SP compared to S, but they exhibited higher Na^+^ permeability values. It was speculated that the introduction of K^+^ would sharply promote the penetration of Na^+^. However, the Na^+^ permeability decreased in SM, which showed that adding Mg^2+^ to the low-sodium salt formulations with relative higher sodium contents may hinder the permeation of Na^+^. Mg^2+^ can strongly combine with the polar groups of proteins and strengthen the interaction between proteins [16], thereby hindering the penetration of Na^+^. Alino et al. [17] also found divalent cations decreased salt penetration into dry-cured ham. In SPMA, SPM, and SP, the K^+^ permeability reaches about 100 times the theoretical value. The reason may be that the charge density of K^+^ is lower than that of Na^+^ and Mg^2+^, making it easy to diffuse in the meat to produce a concentration effect and concentrate at the sampling point. The Mg^2+^ permeability was only about 20% due to the large charge density of divalent ions [18]. No significant difference was observed in the Mg^2+^ permeability of SPMA, SPM, and SM, indicating that Na^+^ and K^+^ had no effect on the penetration of Mg^2+^. 

As shown in Table 1, the weight loss rate of each group basically remained between 45.46% and 55.68%, though the weight loss rate of S (55.68%) was higher than that of other groups; in particular, it was significantly higher than that of SPM (47.42%) and SM (45.46%), indicating that low-sodium salt is beneficial to maintaining the moisture of dry-cured meat products. The lower weight loss of SM may be related to the permeation of ions. As shown in Table 1, SM showed the lowest permeability of Na^+^ and Mg^2+^. The moisture content of dry-cured meat products gradually decreases as the fermentation time progresses, eventually reaching a stable range, and *A*_w_ will also decrease as the moisture content decreases. *A*_w_ affects the nutrition value, color, flavor, and texture of food. In addition, *A*_w_ has an important role in food preservation. As shown in Table 1, the moisture content of each group decreased by about 65% and finally stabilized between 24% and 28%. Although there were no significant differences between the groups, it was noteworthy that the SP had lower moisture content, which was also found in the samples salted with a mixture of NaCl and KCl [17]. As shown in Table 1, the *A*_w_ of all groups was below 0.75, among which the *A*_w_ values of SPMA (0.723) and SPM (0.729) were significantly higher than SP (0.704). In Table 1, it also can be seen that the moisture contents of SPMA, SPM, and SM were higher than that of SP. The higher moisture content was in parallel with a higher *A*_w_ value [17]. Ultimately, the moisture content and *A*_w_ of the Mg^2+^-containing groups (SPMA, SPM, and SM) were higher; Alino, Grau, Toldra and Barat [17] also found the same results.

**Figure 1:**
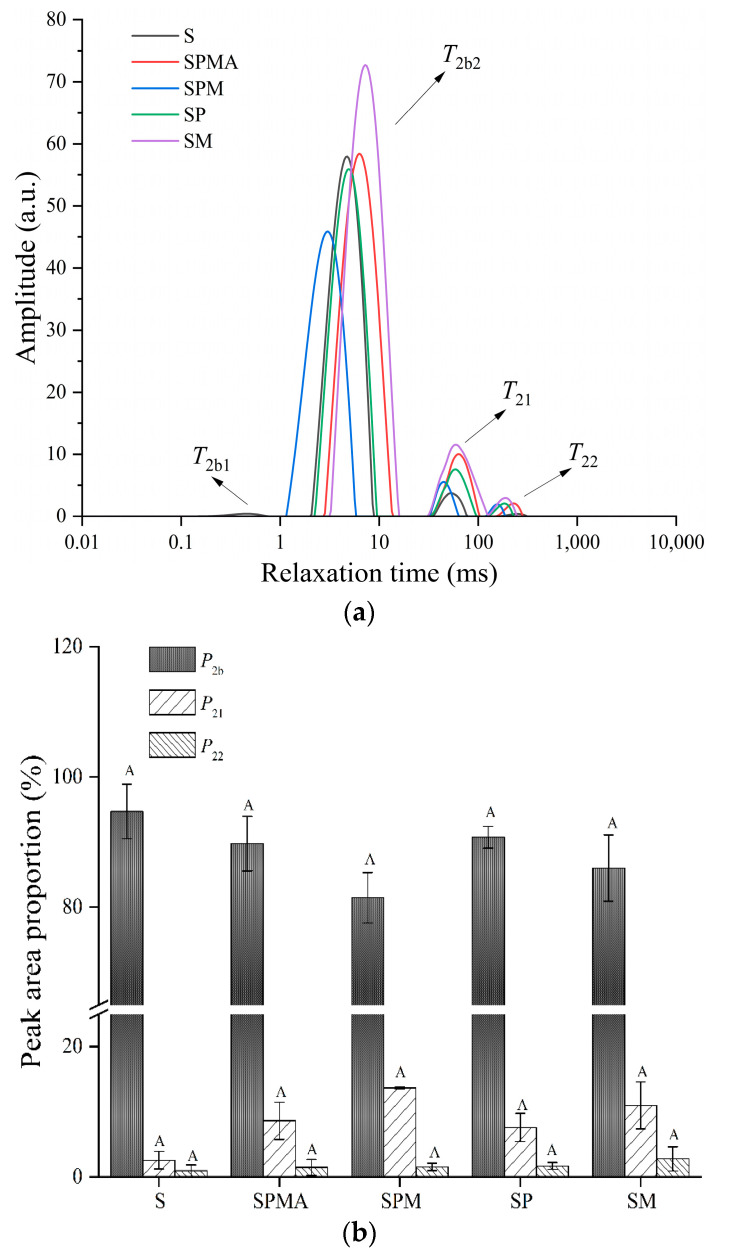
The distribution curves of transverse relaxation time (*T*_2_) (**a**) of peak area proportions of *T*_2b_, *T*_21_, and *T*_22_, (**b**) of dry-cured meat with the addition of different low-sodium salts. S: 100% NaCl; SPMA: 59.375% NaCl+28% KCl+12% MgCl_2_+0.625% L-arginine (Arg); SPM: 60% NaCl+28% KCl+12% MgCl_2_; SP: 72% NaCl+28% KCl; SM: 88% NaCl+12% MgCl_2_. Different letters in the same group indicate significant differences (*p* < 0.05; Tukey test).

Low-field NMR can be used to analyze the distribution and migration of moisture in meat products. The relaxation time *T*_2_ represents the strength of water mobility; that is, a shorter relaxation time represents lower mobility. As shown in Figure 1a, it can be seen that all groups contain three to four relaxation peaks, which has also been reported in the surimi-starch system [19]. The first two peaks were located between 0–1 ms and 1–10 ms, which belong to strongly bound water (*T*_2b1_) and weakly bound water (*T*_2b2_), respectively. The relaxation time between 30–100 ms (*T*_21_) was immobilized water that interacts with the charged groups located between the fiber bundles. The relaxation time greater than 100 ms (*T*_22_) was related to the free water existing outside the myofibril. As shown in Figure 1a, *T*_2b2_ was a major peak in dry-cured meat. The relaxation time of each group was between 3–7 ms. It can be seen from Figure 1b that S had the highest proportion of bound water, reaching 94.69%, while the immobilized water and free water were only 2.56% and 0.94%, respectively. SM enhanced the mobility of bound water, but did not increase the proportion of bound water compared to the S group. Overall, the low-sodium salt groups (SPMA, SPM, SP, and SM) increased the proportions of immobilized water and free water, and reduced the proportion of bound water, so that the dry-cured meat will not be excessively hard and thus be more acceptable to consumers.

#### 2.1.2. pH, Shear Force, and Color

As shown in Table 1, the pH of samples was between 5.90–6.15, which was within a good range of pH value for dry-cured meat products. Among them, the pH values of the low-sodium salt groups (SPMA, SPM, SP, and SM) were slightly lower than those of S. The pH of S (6.11) was significantly higher than SPMA (5.86) and SM (5.90), because the binding of divalent salts to proteins resulted in a decrease in the pH value of the isoelectric point [20].

Muscle is mostly composed of myofibrillar protein, and the effect of myofibrillar protein on tenderness depends not only on the cross-linking degree between protein molecules but also on the interaction between protein molecules. Regarding the shear force, the shear force values of SPMA, SPM, SP, and SM were significantly lower than that of S, which was related to their ionic strength and pH. As shown in Table 1, the pH and ionic strength of the S group were higher, which was beneficial to inhibiting the degradation of muscle fibers, resulting in greater shear force.

Color is one of the main attributes of meat and meat products which reflects freshness and influences consumer purchase intentions. In this study, when sodium was partially replaced by other cations, it had a significant impact on the color parameters, as shown in Table 1. As for L*, S > SPMA > SPM > SM > SP, the L* value of S was significantly higher than that of SP and slightly higher than that of SPMA, SPM and SM. Lorenzo et al. [21] also reported that the L* value of dry-cured bacon salted with a mixture containing KCl in the formula was relatively lower. Mg^2+^ was not contained in SP, indicating that the absence of Mg^2+^ can reduce the brightness of the meat and have an adverse effect on its color. For a*, the order of samples was SPMA > S > SP > SM > SPM. SP was significantly higher than SM and SPM; this is attributed to Arg’s capacity to reduce brown metmyoglobin into bright red oxymyoglobin, improving the quality of dry-cured meat’s a* value [22], which is consistent with the findings of Zhu et al. [23] and Zheng et al. [24]. For b*, S > SPMA > SP > SPM > SM; the b* value of S (26.00) was significantly higher than that of SM (21.72). The presence of Mg^2+^ reduced the yellowness of dry-cured meat, which was good for consumer acceptability.

#### 2.1.3. Effects of Low-Sodium Salt on Fat Oxidation of Dry-Cured Meat 

**Figure 2:**
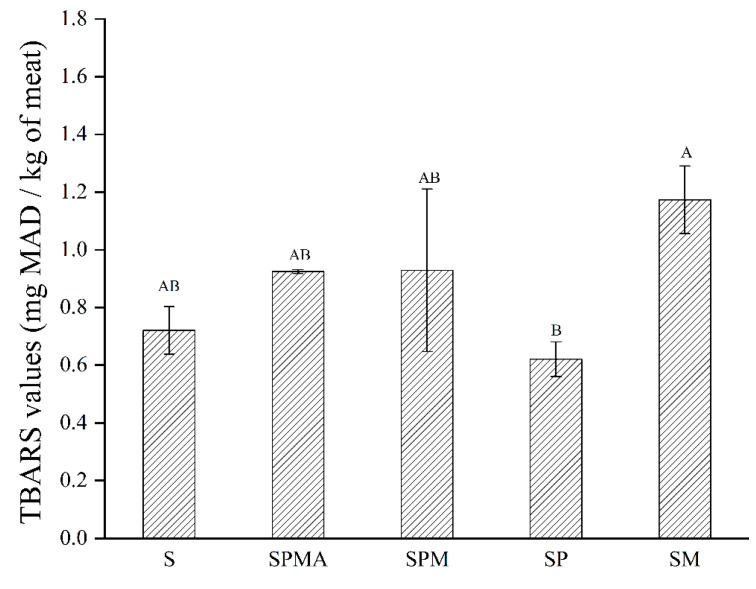
TBARS (Thiobarbituric acid) values of dry-cured meat with the addition of different low-sodium salts. Different letters in the same group indicate significant differences (*p* < 0.05; Tukey test) among treatments. S: 100% NaCl; SPMA: 59.375% NaCl+28% KCl+12% MgCl_2_+0.625% L-arginine (Arg); SPM: 60% NaCl+28% KCl+12% MgCl_2_; SP: 72% NaCl+28% KCl; SM: 88% NaCl+12% MgCl_2_.

The thiobarbituric acid reactive substances (TBARS) value refers to the result of the reaction between derivatives such as thiobarbituric acid and malondialdehyde produced by the oxidative decomposition of unsaturated fatty acids in animal fats and oils. As shown in Figure 2, the TBARS value of SM was significantly higher than that of SP. According to reports in the literature, the TBARS value increases with increasing ionic strength [25]; therefore, pork treated with higher ionic strength mixtures (SPMA and SM) had higher TBARS values, which corresponded to the ionic strength values of each. In a study regarding Brazilian low-sodium dry-fermented sausages containing blends of NaC1, KCl, and CaC1_2_, Dos Santos et al. [26] also reported that the sample treated with a higher ionic strength of 0.7 showed a higher TBARS value.

### 2.2. Effect of Low-Sodium Salts on the Aroma of Dry-Cured Meat

#### 2.2.1. Volatile Compound Profile

The types of volatile organic compounds in raw pork are relatively few, but multiple types of volatile compounds are formed after dry-curing, resulting in a richer flavor. However, the number of volatile compounds in dry-cured meat was still much less than that in dry-cured ham [27]. Compared with dry-cured ham, dry-cured meat had a shorter manufacturing time (around three months versus 12 months). According to Supplementary Table S1, a total of 55 different volatile compounds from nine different chemical groups were identified in the finished dry-cured meat by HS-GC-MS, including 12 aldehydes, 12 esters, 8 alcohols, 8 ketones, 5 alkanes, 2 pyrazines, 4 aromatics, 3 olefins, and 1 furan compounds.

**Figure 3:**
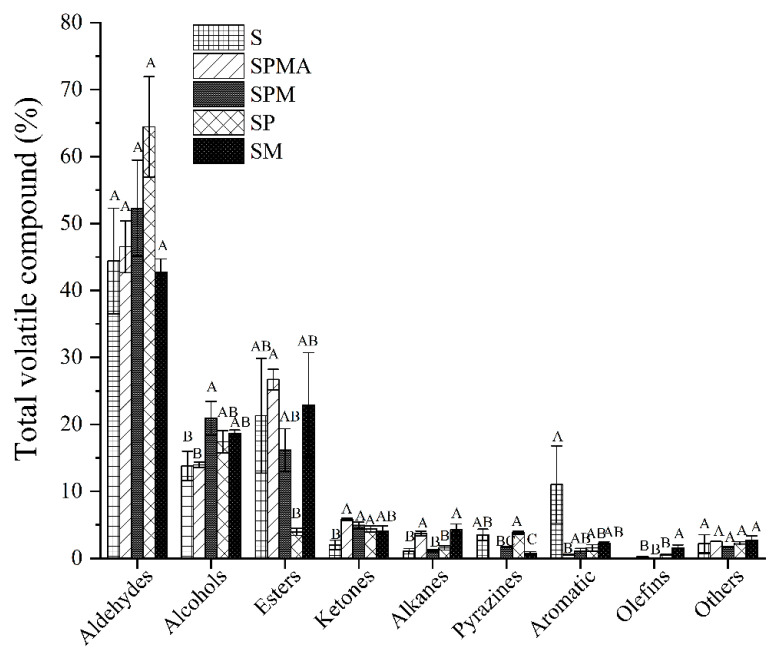
Percentage of various volatile compounds in the total volatile compounds. Different letters in the same group indicate significant differences (*p* < 0.05; Tukey test) among treatments. S: 100% NaCl; SPMA: 59.375% NaCl+28% KCl+12% MgCl_2_+0.625% L-arginine (Arg); SPM: 60% NaCl+28% KCl+12% MgCl_2_; SP: 72% NaCl+28% KCl; SM: 88% NaCl+12% MgCl_2_.

As shown in Figure 3, aldehydes were the most abundant volatile compounds. Aldehyde compounds in each group accounted for 42.49%–64.42% of the total volatiles, among which SP accounted for the highest proportion of 64.42%. The closest proportion to S (46.13%) was SM (42.49%). The order of aldehyde proportion content was SP > SPM > SPMA > S > SM, and there was no significant difference between each group. In addition, SM’s proportions of total volatiles of esters, ketones, aromatics, and furans was also closest to that of S. The proportion of alcohols in each group was in the order of SPM > SM > SP > S > SPMA. The proportion of alcohols in SPM was significantly higher than that in S and SPMA. The proportion of esters in each group was SPMA > SM > S > SPM > SP, and the proportion of esters in SPMA was significantly higher than that in SP. The proportions of ketones and alkanes in each group were SPMA > SPM > SP > SM > S, and SPMA, SPM, and SP were significantly higher than S. The proportion of pyrazines in each group was SP > S > SPM > SM. SP was significantly higher than SM, and SPMA did not contain pyrazines. The proportion of aromatics in each group was S > SM > SP > SPM > SPMA, and S was significantly higher than SPMA. The proportion of furans in each group was SM > SPMA > SP > S > SPM, and there was no significant difference between the groups. In terms of compound types, S, SPMA, SPM, SP, and SM had 34, 39, 41, 38, and 41 volatile compounds, respectively. The findings showed that the addition of low-sodium salt increased the species of volatile compounds in dry-cured meat. Importantly, the SPMA, SPM, and SM groups containing Mg^2+^ had more volatile compounds than SP, which contains KCl.

It was noteworthy that the total concentration of aldehydes was highest in dry-cured meat compared to other compounds, which was consistent with the results in dry-cured ham [28]. It can be seen from Supplementary Table S1 that the total content of aldehydes in all groups showed the following pattern: SP > SM > SPM > SPMA > S, among which the aldehydes content in SP was significantly higher than that of SPMA and S. Among the 12 aldehydes, the OAV values of 3-Methylbutanal, 2-Methylbutyral, Pentanal, Hexanal, Heptaldehyde, Octyl aldehyde, 1-Nonanal, (*E*)-2-Heptanal, (*E*)-2-Octenal, Decyl aldehyde, and (*E*)-2-Nonenal were greater than 1 (Table 2). It is generally accepted that compounds with OAV > 1 play an important role in overall odor [29]. 3-Methylbutanal and 2-Methylbutyral originate from the degradation of amino acids [29], and these compounds contribute positively to the sensory quality of dry-cured meat products, contributing to the overall aroma by providing cheesy, nutty, and salty notes [26]. The order of their content was SM > SPM > SP > S > SPMA and SM > SP > SPM > S > SPMA, respectively. SM was significantly higher than SPMA, because SM had a higher NaCl concentration and thereby promoted the formation of 3-Methylbutanal [30]. It is worth noting that Octyl aldehyde, Hexanal, Pentanal, and 1-Nonanal (linear aldehyde) respectively represent rancid smell, nutty aroma, grassy smell, and strong grease smell. The content of these compounds was relatively high, and the OAV values were all greater than 1, indicating that fat oxidation was active during the dry-curing process of the meat. The content order of each group of these compounds was SP > SM > SPM > SPMA > S, among which SP was significantly higher than S. These compounds usually originate from fat autooxidation reactions [31,32], and adding low-sodium salt will increase the content of the aforementioned linear aldehydes because low-sodium salt promotes fat oxidation. It was speculated that the pro-oxidative activity of lower concentrations of NaCl may be further enhanced due to the presence of K^+^ [11]. (*E*)-2-Heptanal and (*E*)-2-Nonenal had a green, fatty, and rancid taste, but there were no significant differences between the groups. Decyl aldehyde has a spicy waxy odor and is formed by β-oxidation of unsaturated fatty acids [33]. The Decyl aldehyde content in SP was significantly higher than that in SPMA and S, which may be explained by the fact that the presence of KCl enhances the β-oxidation reaction [33]. (*E*)-2-Octenal exhibited a sweet aroma or the smell of cucumber or melon. The (*E*)-2-Octenal in SP and SPM was significantly higher than that in SPMA, SM, and S, because SM contains more diketone compounds and amino acids (Table 3 and Table S1), while the formation of (*E*)-2-Octenal requires free amino acids and diketone compounds. 

Alcohols often have aromatic, vegetal, and earthy notes and are an integral part of dry-cured cuts of meat. A total of 8 alcohols were detected in the meat, and the order of the total content of each group was as follows: SM > SP > SPM > SPMA > S, among which SM, SP, and SPM were significantly greater than SPMA and S, indicating that the low-sodium group without Arg addition significantly increased the alcohol content of dry-cured meat, because NaCl itself has antibacterial activity, whereas partial substitution will result in stronger microbial growth and higher branched-chain alcohol production [34]. Among the eight alcohols, the OAVs of 3-Methyl-1-butanol, 1-Pentanol, 1-Hexanol, 1-Octen-3-ol, 1-Heptanol, and 1-Octanol were all greater than 1. No significant difference was observed in each group for alcohols containing 3-Methyl-1-butanol, 1-Pentanol, 1-Hexanol, and 1-Octen-3-ol. 1-Heptanol has a strong aromatic odor, and the order of content in each group was SM > SP > SPM > S > SPMA, with SM being significantly higher than the other groups. 1-Octanol has a pungent odor, which may come from the oxidation of myristic acid, linoleic acid, palmitoleic acid, or oleic acid. The order of the content of each group was SP > SM > SPM > SPMA, among which SP was significantly higher than SPMA. There was no Octanol in S, probably because the high content of NaCl inhibited the growth of microorganisms and reduced the production of branched-chain alcohols.

Ester compounds are derived from the esterification reaction of carboxylic acids and alcohols. Esters containing short-chain acids have a fruity flavor, but long-chain acids produce fatty flavors [28]. The order of the total content of esters in each group was SM > SPMA > S > SPM > SP. SM was significantly higher than SP, indicating that partial replacement of magnesium increases the content of esters in dry-cured meat. Among them, the OAVs of Methyl butyrate, Methyl 2-methylbutyrate, Methyl Isovalerate, Methyl pentanoate, Methyl caproate, Ethyl caproate, γ-Caprolactone and γ-octa lactone were higher than 1, and there was no significant difference between the groups in the contents of Methyl Isovalerate, γ-Caprolactone, and γ-octa lactone. The most abundant ester in each group was methyl hexanoate, which has a pleasant smell and comes from the esterification reaction of ethanol and acid. The order of methyl hexanoate content in each group was SM > SPMA > S > SPM > SP, among which SM was significantly higher. Methyl butyrate has an apple aroma, and the content order of each group was SM > SPMA > SPM > S > SP. Methyl 2-methylbutyrate ester has a pungent, ethereal fruity smell, and the content order of each group was SM > S > SPM > SPMA > SP. Ethyl caproate has a fruity aroma like pineapple, and the content order of each group was as follows: SM > SPM > SPMA > S. The levels of Methyl butyrate, Methyl 2-methylbutyrate, and Ethyl caproate in SM were significantly higher than in the other groups.

Ketone compounds are produced when alkoxy groups are oxidized by alkyl radicals during lipid oxidation to generate ketone compounds and hydrocarbons. The order of the total content of ketones in each group was as follows: SM > SP > SPMA > SPM > S. The total content of ketones in S was significantly lower than that of other groups, indicating that the addition of low-sodium salt increased the amount of ketones in dry-cured meat. Among them, the OAVs of 2-Heptanone and 6-Methyl-5-hepten-2-one were greater than 1, and 6-Methyl-5-hepten-2-one has been considered a safe compound [35], but there was no significant difference between the contents of each group. 2-Heptanone is an oxidation product of fatty acids [30]. It has a fruity aroma similar to pear and has been detected in many dry-cured meat products, such as ham and sausages [30]. The order of the content of each group of 2-Heptanone was SM > SP > SPMA > SPM > S, where SM was significantly higher than SPM and S, among which the origin of linear 2-ketones was related to the β-oxidation activity of mold growing on the surface of dry-cured meat products [36]. The reason may be that the high ionic strength of SM enhances fat oxidation of dry-cured meat [26].

As for alkane compounds, there were very few types and contents in dry-cured meat. In addition, the threshold value was high, so their contribution to the aroma of the meat was very small. As shown in Table 2, among the alkane compounds, there was no OAV value greater than 1. Our study found that 2,3,5-Trimethylpyrazine and Tetramethylpyrazine had OAVs greater than 1. The order of the total content of pyrazine substances in each group was SP > S > SPM > SM, but SPMA does not contain pyrazines. 2,3,5-Trimethylpyrazine and Tetramethylpyrazine have the smell of corn, nuts, hazelnuts, baking, spices, and chocolate [37], and are usually formed by the condensation of two α-aminocarbonyl groups through the Strecker reaction. The order of content in each group was SP > S > SM > SPM and SP > S > SPM, respectively, among which SP was significantly higher than the other groups. It can be formed from free amino acids and reducing sugars [38]. Four aromatic compounds were also discovered in SM. The total amount in S was the highest, but there was no significant difference between the groups. In addition, three olefin compounds were found, namely 1-Octene, trans-2-Octene, and Dipentene, but there was no significant difference between the groups. Finally, a furan compound was discovered. Furan compounds provide a lot of aromas to meat products. 2-Pentylfuran is a linoleic acid oxidation product with the sensory properties of beans and green grass and a low threshold [39]. SM had the highest 2-Pentylfuran content, but there was no significant difference between the groups. 

#### 2.2.2. Aroma in Dry-Cured Meat Determined by Electronic Nose 

**Figure 4:**
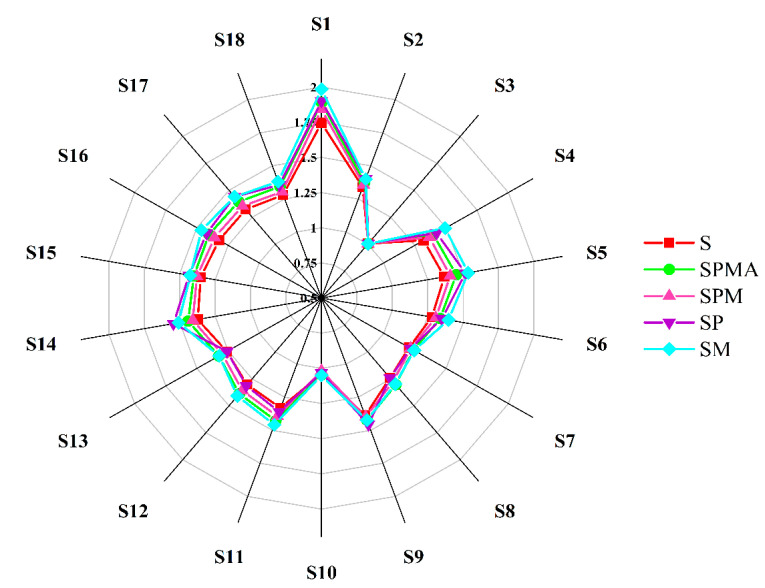
Radar chart of electronic nose analysis for dry-cured meat with the addition of different low-sodium salts. S: 100% NaCl; SPMA: 59.375% NaCl+28% KCl+12% MgCl_2_+0.625% L-arginine (Arg); SPM: 60% NaCl+28% KCl+12% MgCl_2_; SP: 72% NaCl+28% KCl; SM: 88% NaCl+12% MgCl_2._

The electronic nose radar chart intuitively shows the response intensity to various substances. Figure 4 shows the results of the response intensity of volatile substances produced by dry-cured meat under 18 types of sensors. The response substances corresponding to the 18 types of sensors are S1 (Alkanes, smoke), S2 (alcohols, aldehydes, short-chain alkanes), S3 (ozone), S4 (sulfides), S5 (organic amines), S6 (organic gases, benzophenones, aldols), S7 (short-chain alkanes, such as methane, natural gas, and biogas), S8 (short-chain alkanes, such as propane and liquefied gas), S9 (aromatic compounds, aldols), S10 (hydrogen-containing gas), S11 (alkanes, alkenes), S12 (short-chain alkanes, such as liquefied gas and methane), S13 (combustible gases, such as methane), S14 (combustible gases and smoke), S15 (alkanes, organic gases), S16 (sulfides), S17 (nitrides), S18 (ketones, alcohols). As illustrated in Figure 4, the response values of S1, S4, S5, S6, S7, S10, S11, S12, S16, S17, and S18 sensors in SM were all the highest, indicating that SM contained more volatile compounds, which was consistent with the results of GC-MS (Supplementary Table S1).

### 2.3. Effect of Low-Sodium Salts on the Taste of Dry-Cured Meat

#### 2.3.1. Molecular Weight Distribution of Peptides

**Figure 5:**
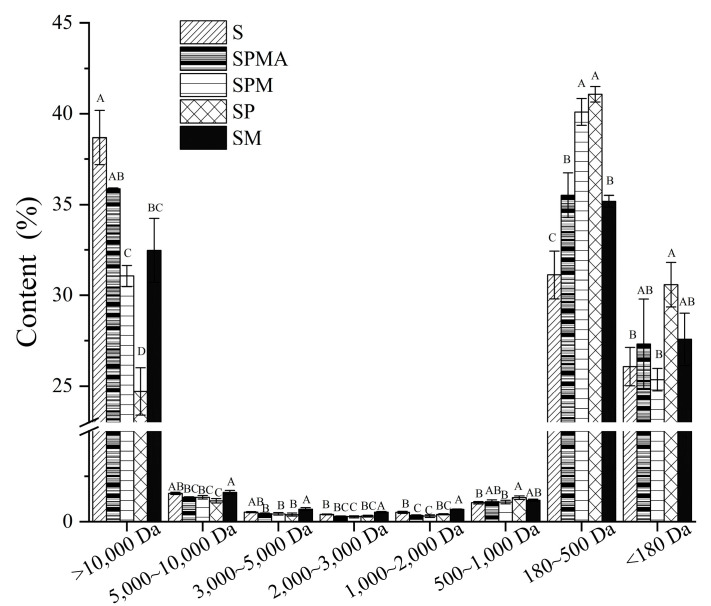
Molecular weight distribution in dry-cured meat with the addition of different low-sodium salts. Different letters in the same group indicate significant differences (*p* < 0.05; Tukey test) among treatments. S: 100% NaCl; SPMA: 59.375% NaCl+28% KCl+12% MgCl_2_+0.625% L-arginine (Arg); SPM: 60% NaCl+28% KCl+12% MgCl_2_; SP: 72% NaCl+28% KCl; SM: 88% NaCl+12% MgCl_2._

The distribution of peptide molecular weight can reflect protein degradation. As shown in Figure 5, the molecular weights of the peptides in each group of dry-cured meats are mainly distributed in three ranges: molecular weights greater than 10,000 Da, 180–500 Da, and less than 180 Da. Peptides with a molecular weight greater than 10,000 Da include some macromolecular peptides or preliminarily hydrolyzed proteins [40]. The peptide molecular weight (>10,000 Da) in each group followed the order S > SPMA > SM > SPM > SP; in particular, S was significantly higher than SPM, SP, and SM, indicating that partial substitution of sodium facilitates protein degradation. The peptides with a molecular weight distribution between 180 and 500 Da may be related to polypeptides that produce flavor, salty taste, and other tastes. The order of those in each group was SP > SPM > SPMA > SM > S, among which SP and SPM were significantly higher than other groups. For molecular weights less than 180 Da, related to the content of free amino acids, the contents of SP, SM, and SPMA were greater than those of S and SPM, which implies that more free amino acids formed in SP, SM, and SPMA.

#### 2.3.2. Free Amino Acid Contents in Dry-Cured Meat

The release of free amino acids (FAAs) results from protein degradation. As shown in Table 3, the promoting effect on total free amino acids (TFAAs)accumulation of KCl and MgCl_2_ partially replacing NaCl was consistent with the results of Armenteros et al. [41] and José M et al. [42]. The TFAAs content of SM was the highest at 2391.7 mg/100g, which was significantly higher than that of S and SPM, which is related to their pH. Aksu et al. [43] reported that low pH could increase protein degradation. Table 1 also show that SM with higher TFAAs had lower pH than S and SPM.

The umami amino acids in SM were significantly higher than those in the other groups, indicating that partial replacement of NaCl with MgCl_2_ can promote protein degradation to produce more Glu and Asp. In addition, Kęska et al. [44] found that Glu can impart salty and umami flavors to dry-cured meat and inhibit bitterness. The sweet amino acid content of SP was significantly higher than that of the other four groups, which indicates that only KCl can produce the formation of more sweet-related amino acids when it partially replaces NaCl. For bitter amino acids, the total content order was SM > SPM > SPMA > SP > S. It can be seen that the low-sodium salt groups have more bitter amino acids, but they are all within the appropriate range. In the present study, the final free amino acid content was lower than that reported by the authors of [45] in dry-cured hams, which may be due to shorter processing times.

#### 2.3.3. Taste Analysis by Electronic Tongue

**Figure 6:**
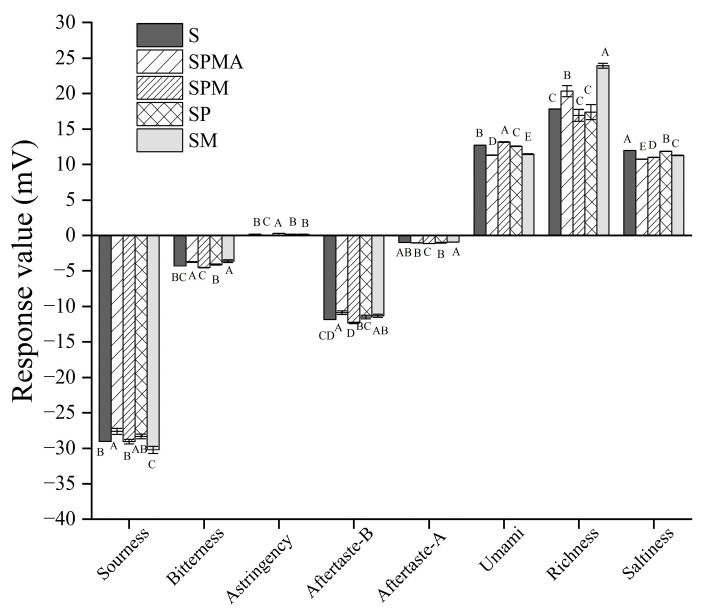
Electronic tongue analysis of dry-cured meat with different low-sodium salt additions. Different letters in the same group indicate significant differences (*p* < 0.05; Tukey test) among treatments. S: 100% NaCl; SPMA: 59.375% NaCl+28% KCl+12% MgCl_2_+0.625% L-arginine (Arg); SPM: 60% NaCl+28% KCl+12% MgCl_2_; SP: 72% NaCl+28% KCl; SM: 88% NaCl+12% MgCl_2._

In addition to affecting the aroma, low-sodium salt also affects the taste. Therefore, an electronic tongue was used to measure the saltiness, bitterness, sourness, and richness of dry-cured meat. In terms of richness, the response values order was SM > SPMA > S > SP > SPM (Figure 6). It is worth noting that comparing SPMA and SPM revealed a small difference in Arg, but their richness was significantly different, indicating that adding a small amount of Arg could increase the richness of dry-cured meat. The response values of umami taste in each group were in the following order: SPM > S > SP > SM > SPMA. Conversely, the umami taste intensity of SPMA was significantly lower than that of SPM. These results show that adding Arg cannot improve the umami taste of dry-cured meat, but has an inhibitory effect. 

In terms of salty attributes, the response values of each group were SM > SP > S > SPM > SPMA, which was not consistent with the sodium ion content shown in Table 1. This showed that the salty attributes were not only produced by Na^+^ but can also be produced by other metal ions as well as peptides and free amino acids. As shown in Figure 5, the amount of peptides of molecular weight 180–500 Da in SM and SP was significantly higher than that in other groups, indicating that Mg^2+^ or K^+^ alone can promote the production of salty peptides. As for bitterness, the response values were SM > SPMA > SP > S > SPM, among which SM and SPMA were significantly higher than SP, S, and SPM. Zhang et al. [46] also found that the presence of KCl and MgCl_2_ enhanced the bitter taste. However, the bitterness values of each group in this study were less than the lowest response value of 4.91, which was acceptable to the senses. In terms of sourness, although there were differences in the response values between samples, the values were all less than the minimum response threshold of −13, which can be considered to be extremely weak sourness.

### 2.4. Effect of Low-Sodium Salts on Sensory Scores of Dry-Cured Meat

The dry-cured meat was evaluated in four areas: appearance, taste, texture, and overall acceptance, with scores ranging from 1 to 5. As shown in Table 4, SM received the highest scores in gloss, saltiness, umami, and fat adhesion. Its bitterness was the lowest among the low-sodium salt group. Its overall acceptance was also the highest among the low-sodium salt group, indicating that the sensory evaluators were more satisfied with SM. In terms of color, the colors of the low-sodium salt groups (SPMA, SPM, SP, and SM) were all redder than S, with SP having the highest redness, which was consistent with the color results shown in Table 1. The bitterness scores of the low-sodium salt group were higher than those of S, which was consistent with the content of bitter amino acids shown in Table 3. According to reports, substitution of 50% KCl increases the bitterness of dry-cured meat [10], but addition levels of 28% and 12% (KCl and MgCl_2_) did not result in reports of bitterness from the assessors in our study. In terms of umami taste, SM scored the highest, which was consistent with the umami amino acid content shown in Table 3. As shown in Figure 2, SM had a higher TBARS value. It was speculated that SM had the highest degree of fat oxidation, resulting in higher adhesion. The overall acceptance scores of SM and SPMA were the highest among the low-sodium salt groups.

### 2.5. Principal Component Analysis (PCA) of Physicochemical Properties, Aroma, Taste, and Sensory Scores

**Figure 7:**
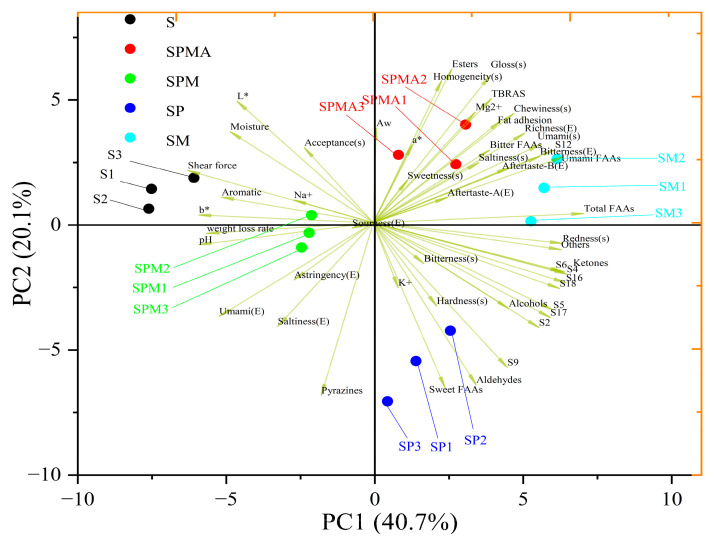
PCA of physicochemical properties, aroma, taste, and sensory scores. S: 100% NaCl; SPMA: 59.375% NaCl+28% KCl+12% MgCl_2_+0.625% L-arginine (Arg); SPM: 60% NaCl+28% KCl+12% MgCl_2_; SP: 72% NaCl+28% KCl; SM: 88% NaCl+12% MgCl_2_.

PCA can reduce the dimensionality of complex data and further reflect the overall information provided by the sample. In Figure 7, PC1 and PC2 cover 41.8% and 19.5%, respectively, with a cumulative value of 61.3%. The five treatment groups in this study were clearly separated in terms of PC1. Furthermore, SP differed from other components by PC2 through indicators such as sweet amino acids, aldehydes, pyrazines, shear force, and K^+^ content. Most physicochemical properties were grouped in the negative quadrant of PC1, whereas most sensory attributes, electronic nose odor, and free amino acid content were grouped in the positive quadrant of PC1. The components detected by GC-MS, especially aldehydes, ketones, alcohols, and pyrazine, have a strong correlation with the odor detected by electronic nose. S was described as related to shear force, aromatic compound content, Na^+^ content, and b*. However, excessive shear force and pH are detrimental to meat products. SM was described as related to electronic tongue aftertaste, free amino acids, total amino acids, and electronic tongue bitterness. SM and S were the farthest apart, indicating a significant difference between them, which was conducive to the enhancement of the quality of dried-cured meat in terms of shear force, water loss, and pH. In addition, SPMA was considered to be related to *A*_w_, ester content, TBARS, Mg^2+^ content, sensory properties, and bitter amino acids. Also, SP was described as related to electronic nose S9, sensory hardness, sweet amino acid content, aldehyde content, and K^+^ content.

Importantly, SPMA, SPM, and SM containing Mg^2+^ had more volatile compounds than SP in terms of flavor (Figure 3). Sensory evaluation also found that SM had a higher overall acceptance compared to other groups. Therefore, magnesium ions contribute to improving the quality of dry-cured meat products.

## 3. Materials and Methods

### 3.1. Materials

Fresh pig hind legs were purchased from a local supermarket in Kunming, Yunnan Province. Food-grade magnesium chloride (NaCl), potassium chloride (KCl), magnesium chloride (MgCl_2_), and L-arginine (Arg) were obtained from Henan Mingrui Food Additives Co., Ltd. (Zhengzhou, China). Trichloroacetic acid and thiobarbituric acid were obtained from Shanghai Titan Technology Co., Ltd. (Shanghai, China). Analytical grade KCl was sourced from Yunnan Jingrui Technology Co., Ltd. (Kunming, China). Sodium dihydrogen phosphate (NaH_2_PO_4_), disodium hydrogen phosphate (Na_2_HPO_4_), ice acetic acid (HAC), dimethyl sulfoxide (DMSO), and sodium acetate (NaC_2_H_3_O_2_) were obtained from Tianjin Zhiyuan Chemical Reagent Co., Ltd. (Tianjin, China). Triton X-100 was obtained from Beijing Solarbio Science & Technology Co., Ltd. (Beijing, China). Chloroform (TCM) and concentrated nitric acid (HNO_3_) were purchased from Yonghua Chemical Co., Ltd. (Suzhou, China). Dithiothreitol (DTT) was obtained from Beijing Solabao Technology Co., Ltd. Z-Phe-Arg-AMC and Z-Arg-Arg-AMC were sourced from Shanghai Zhenzhun Biotechnology Co., Ltd. Disodium EDTA was purchased from Guangzhou Saiguo Biotechnology Co., Ltd. (Guangzhou, China). Brij 35 was obtained from Shanghai Maclin Biochemical Technology Co., Ltd. (Shanghai, China). All reagents mentioned above, except for the food-grade ones, were of analytical grade.

### 3.2. Preparations of Dry-Cured Meat

**Figure 8:**
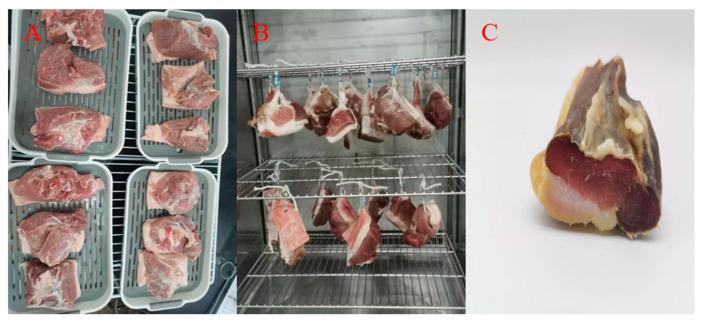
The appearance of meat (**A**) during curing, both in the (**B**) early and (**C**) later stage of natural fermentation.

The hind leg of the pig was uniformly cut into cubes with a weight of 500 g. Five formulas of salt were employed to cure the meat according to common low-sodium salt formulas from Finland with minor modifications [47]. The salt (5%, based on the weight of the meat) was placed on the surface of the meat and rubbed by hand to accelerate the permeation of the salt. The meat was dry-cured for 6 days at 4 °C and a relative humidity (RH) of 80%–90% before drying at 12 °C and an RH of 50% for 3 days in a constant temperature and humidity chamber (ICTHI-1100T, STIK, Stochastic Instruments Co., Ltd., Shanghai, China). For subsequent fermentation, the sample was divided into 4 stages including the early fermentation stage (20 °C, RH 60%, and 15 days), middle fermentation stage (28 °C, RH 75%, and 70 days), and late fermentation stage (20 °C, RH 65%, and 5 days). Digital images of dry-cured meat in the experiment were shown in Figure 8. Each group consisted of 3 parallel samples. The five formulas comprise one control group and four groups of low-sodium salts.

(1) S (control): 100% NaCl, ionic strength 1.160 mol/g;
(2) SPMA: 59.375% NaCl+28% KCl+12% MgCl_2_+0.625% L-arginine (Arg), ionic strength 1.160 mol/g;
(3) SPM: 60% NaCl+28% KCl+12% MgCl_2_, ionic strength 1.085 mol/g;
(4) SP: 72% NaCl+28% KCl, ionic strength 1.055 mol/g;
(5) SM: 88% NaCl+12% MgCl_2_, ionic strength 1.190 mol/g.

### 3.3. Sodium, Potassium, and Magnesium Content

The metal ion content in the various treated meats was determined according to national standards [48,49] with minor modification, using atomic absorption spectroscopy (Mars 6, American CEM Service Corp, Charlotte, NC, USA). The chopped sample (0.5 g) was added into 10 mL of HNO_3_ for digestion using a microwave digestion system (EHD-24, Donghang Scien-Tech, Beijing, China). After cooling down, the mixture was placed on an electric heating oven to drive HNO_3_ to near dryness, and adjusted to a constant volume of 50 mL with deionized water, immediately followed by the determination of sodium, potassium, and magnesium contents by atomic absorption spectrometry. The permeability was calculated based on the difference between the actual ion content and the theoretical value. The standard curves were established based on standard substances for sodium, potassium, and magnesium ions. The standard curve equations for calculating the content of sodium, potassium, and magnesium were *y* = 0.3728*x* + 0.0298, *R*^2^ = 0.9972; *y* = 02277*x* + 0.016, *R*^2^ = 0.9944; *y* = 0.6554*x* + 0.4554, *R*^2^ = 0.9916, respectively. The sodium, potassium, and magnesium contents remaining in the meat and the permeability were calculated by the following equations:

$$X=\frac{C\times V\times f}{100\times m\times \left(1-w\right)}$$

$$P=\frac{L-X}{X}\times 100\%$$

where *X* is the salt content (g/100 g dry weight); *C* is the sodium content obtained from standard curves (mg/mL); *V* is the solution volume (mL); *f* is the dilution rate; *m* is the sample weight (g); and *w* is the moisture content. *P* is permeability (%); *L* is theoretical addition amount, that is, the amount of added metal ions.

### 3.4. Weight Loss

At the end of fermentation, the meat was weighed and its weight recorded. The weight loss was calculated using the following equation:

$$Weight\;loss\;(\%)=(1-\frac{Weight\;of\;meat\;at\;the\;end\;of\;fermentation\left(g\right)}{Fresh\;meat\;weight\left(g\right)})\times 100\%$$

### 3.5. Moisture Content and Water Activity (A_w_)

The moisture content of dry-cured meat was assessed by the weight difference of meat before and after drying at 105 °C. 

To determine the water activity (*A*_w_), the chopped sample (2.0 g) was tiled in a water activity measuring dish so that the sample completely covered the bottom of the cassette. Then, the cassette was placed in a pre-heated water activity meter (HD-3A-4, Nanjing, China) for 20 min for water activity determination.

### 3.6. Low-Field ^1^H NMR Measurements

A low-field nuclear magnetic resonance analyzer (LF-NMR) with a proton resonance frequency of 49.74 MHz and a permanent magnet strength of 20 ± 0.08 T was used to analyze the water state in dry-cured meat according to the method reported by Wang et al. [50] with some modifications. A quantity of meat with the same weight (4.0 g) was placed in a 30 mm diameter NMR tube, and signals were acquired using a CPMG pulse sequence. The main parameters were set as follows: TW (wait time) of 3000 ms, TE (echo time) of 1.0 ms, NECH (number of echoes) of 1000, and NS (number of scans) of 4. MultiEx Inv analysis software (NIUMAG nuclear magnetic resonance analysis application software Ver4.0) was used to fit the CPMG decay curve with a multi-exponential fitting and the SRIT algorithm was used to obtain the transverse relaxation time (*T*_2_) curve and the corresponding NMR parameters.

### 3.7. pH

The meat (10 g) was put into a 150 mL centrifuge tube, containing 100 mL of 0.75% KCl solution. The mixture was homogenized for 1 min using a homogenizer (FS-2, Guoyu Instruments, Changzhou, China) and filtered with a gauze. The pH of the filtrate was measured immediately using a pH meter (PHS-3C, Yidian Scientific Instruments Co, Shanghai, China).

### 3.8. Shear Force

The method was slightly modified from that described by Alino, Grau, Toldra and Barat [17]. The meat was cut into a 200 mm × 200 mm × 200 mm cube and sheared on a texture analyzer (TA.XT. Plus, Stable Micro Systems, Godalming, UK) equipped with a load cell of 50 kg. The testing conditions were as follows: the pre-test probe speed was 2 mm/s, the test speed was 1 mm/s, and the post-test speed was 2 mm/s. The trigger force and compression distance were set as 5 g and 28 mm, respectively. The probe type used was HDP/BSK.

### 3.9. Surface Color

The color of the dry-cured meat was measured using a colorimeter (CR-400, Konica Minolta, Tokyo, Japan) with a D65 light source. The colorimeter was calibrated with a standard whiteboard (ch00) and blackboard before measurement. The probe with a lighting section diameter of Φ 8mm lightly touched the meat pieces, which were sliced to a thickness of about 3–5 mm. The observation angle was approximately 2°. The color was expressed in terms of luminosity (L*), redness (a*), and yellowness (b*) values. Each measurement was performed in triplicate.

### 3.10. Thiobarbituric Acid (TBA) Values

Proportional amounts of lean and fat meat samples (2 g) were weighed and mixed with 17 mL 2.5% trichloroacetic acid (TCA) and 3 mL 1% thiobarbituric acid (TBA). After heating in a boiling water bath for 30 min, the mixture was removed and cooled to room temperature. Four milliliters of the supernatant were mixed with an equal volume of chloroform, shaken to mix thoroughly, and centrifuged at 3000 rpm for 10 min at room temperature. The absorbance of the supernatant was measured at 532 nm after calibration with chloroform.

### 3.11. Volatile Compound Measurement

The extraction of volatile compounds was carried out using the headspace solid-phase microextraction (HS-SPME) technique [51]. A chopped sample of 3.00 g was added into a 20 mL headspace vial. 1,2-dichlorobenzene (100 mg/L) of 4 µL was added as an internal standard, and the vial was sealed with a polytetrafluoroethylene (PTFE) silicone septum. The vial was then equilibrated for 10 min in a water bath at 45 °C. A divinylbenzene/carboxy/polydimethylsiloxane (DVB/CAR/PDMS) fiber was selected, exposed in the headspace of the vial, and extracted for 30 min at 45 °C. Finally, gas chromatography-mass spectrometry (GC-MS) was used for qualitative and quantitative analysis. A DB-wax (0.25 mm × 0.25 µm × 30 m) capillary column was used. The inlet temperature was 250 °C, and the SPME fiber was desorbed into the GC inlet for 5 min. The split ratio was set as 1:5. The temperature program of the oven was as follows: initial temperature of 40 °C, held for 2 min, increased to 90 °C at a rate of 3 °C /min, held for 5 min, then increased to 200 °C at a rate of 3 °C /min and to 230 °C at a rate of 15 °C /min, held for 10 min. Helium was used as the carrier gas at a flow rate of 1.5 mL/min. The interface temperature was 280 °C, and the ion source temperature was 230 °C. Electron energy was 70 eV. The scan mode was used with a mass scan range of 45~450 *m*/*z*. Compounds were identified by searching the NIST 21 mass spectral library and retention indices (RI), with a similarity > 90%. The relative content of each compound was calculated by comparing its area to that of the internal standard. Three parallels were set for each treatment, and the results were calculated on a dry basis and further analyzed by taking the average value. The OVA value was calculated according to the following equation:

$$\mathrm{OAV}=\frac{C}{T}$$

*C* is compound content (μg/kg); *T* is the threshold value for the compound.

### 3.12. Electronic Tongue Detection

Peptide molecular weight was determined according to the method of Wang et al. [52]. The sample (25 g) was mixed with 100 mL of ultrapure water and homogenized using a homogenizer (T25, IKA, Staufen, Germany). The suspension was filtered using a filter paper, and then the collected filtrate was centrifuged at 4 °C and 8000 r/min for 10 min. The supernatant was filtered again using filter paper. The richness, saltiness, sourness, umami, bitterness, astringency, aftertaste-B, and aftertaste-A of the sample were analyzed using an electronic tongue (SA402B, INSENT, Kanagawa, Japan).

### 3.13. Peptide Molecular Weight Distribution Determination

Peptide molecular weight was determined according to the method of Zhan et al. [53], which was modified by Wang et al. [52]. An accurately weighed 3 g of chopped sample was mixed with 30 mL of ultrapure water, and homogenized at 10,000 r/min for 30 s in an ice bath. Subsequently, the mixture was centrifuged at 8000 r/min and 4 °C for 10 min. Ultimately, the supernatant was filtrated with a double-layer filter paper, and then a 0.22 μm PES filter membrane before measurement.

### 3.14. Free Amino Acid Measurement

A high-performance liquid chromatography (HPLC) method was used to detect the content of free amino acids in the dry-cured meat [52]. Firstly, 1 g of meat sample was accurately weighed and mixed with 25 mL of 5% TCA, followed by 30 min of ultrasonic treatment. After standing for 2 h, the suspension was centrifuged at 10,000× *g* for 10 min. Finally, the supernatant obtained after centrifugation was filtered through a 0.45 μm PES filter membrane. The chromatographic conditions were as follows: an ODS Hypersil column (250 mm × 4.6 mm × 5 μm) was used, and the column temperature was kept at 40 °C. The mobile phases A and B were 0.6 mmol/L sodium acetate and 0.15 mmol/L sodium acetate-acetonitrile-methanol (1/2/2, *v*/*v*/*v*), respectively. The flow rate was set to 1.0 mL/min.

### 3.15. Electronic Nose Detection

The response of volatile compounds was measured using an electronic nose (C-Nose, Baosheng Industrial Development Co., Ltd., Shanghai, China) consisting of 18 metal oxide gas sensors (S1–S18). The minced meat sample (4 g) was placed in a 20 mL headspace vial, and equilibrated at 50 °C for 50 min. The cleaned sensor array was set to zero with processed clean air as the carrier gas. Then, 200 µL of the sample was injected with a wash time of 100 s and a test time of 60 s, and the device was cleaned for 5000 s before each measurement. Three measurements were conducted for each sample.

### 3.16. Sensory Analysis

Selection of sensory assessors: fifty volunteers aged 20–25 years old were recruited from Kunming University of Science and Technology. According to National standard [54], a questionnaire survey was designed to understand the interests and motivations of sensory assessors, whether they dislike dry-cured meat products, whether they have taken drugs that impair sensory function or worn dentures, and whether there is sufficient time. Subsequently, the volunteers were trained in terms of identification and intensity discrimination for taste, aroma, texture, and color attributes. The training program was conducted over 4 stages in one month. Ultimately, 18 assessors (9 males and 9 females) were selected by identification and intensity tests of taste, aroma, texture, and color attributes.

According to the method described by Sugimoto et al. [55], the quality of dry-cured meat was evaluated in 5 attributes including appearance, taste, texture, and overall acceptance. Each sensory assessor evaluated the sample on a scale of 1 to 5. The quality of the meat was judged on a scale of 1 to 5. Table 5 shows the sensory evaluation criteria. 

Before analysis, the sample was sliced with a thickness of 2 mm, and then cooked at 90 °C in a water bath. The entire slice, including lean and fat areas, was placed on a plastic board encoded with a 3-digit number and presented to the assessors. The order of sample presentation systematically varies between evaluators to balance the impact of service order and carryover. During the evaluation period, the assessors were provided with salt-free biscuits and water for cleaning the oral cavity. All tests were conducted in a standard sensory laboratory. The sensory testing was evaluated by the ethical committee of Kunming University of Science and Technology.

### 3.17. Statistical Analysis

Unless otherwise stated, three parallel experiments were set up in each group. Experimental data were processed by one-way ANOVA in Minitab 21, and results were expressed as mean ± standard deviation (SD). Multiple comparisons were made by the Tukey test, with *p* < 0.05 indicating a significant level of difference. 

To assess the contribution of the major physicochemical parameters, volatile compounds, electronic nose data, molecular weight distribution, free amino acids, electronic tongue data, and sensory scores in all samples, the data were evaluated by multivariate principal component analysis (PCA) using the origin 2022.

## 4. Conclusions

Low-sodium salt-cured meat products have good development prospects and are suitable for the needs of people with low sodium intake. In this study, four groups of low-sodium salts (SPMA, SPM, SP, and SM) and one group of pure NaCl (S) were used to process meat, and their physical and chemical properties, aroma, and taste were analyzed. It was found that Mg^2+^ can strongly combine with the polar groups of proteins to strengthen the interaction between proteins, thus hindering the penetration of salt. The Na^+^ permeability of the SM was only 77.85%, which was much lower than other groups, and the salt reduction reached 24.19%. Regarding flavor, partial replacement of metal ions, except for SP, promoted the oxidation of meat and increased the types of volatile compounds. In addition, the low-sodium salt groups had lower pH values. A low pH value will increase the degradation of protein, causing more proteins in the low-sodium salt groups to degrade into small molecule peptides and amino acids, making dry-cured meat products richer in taste. The study provides a clearer direction for further exploration of marinade recipes and provides a theoretical basis for low-sodium dry-cured ham.

## Figures and Tables

**Table 1:** Physical and chemical properties of dry-cured meat with the addition of different low-sodium salts.

Type	Index	Groups				
		S	SPMA	SPM	SP	SM
Metal ion contents and permeability (dry basis)	Na^+^ (%)	5.63 ± 0.22 ^A^	3.70 ± 0.29 ^D^	3.48 ± 0.16 ^D^	4.11 ± 0.17 ^C^	4.64 ± 0.25 ^B^
	Theoretical value	6.77	4.04	4.06	4.88	5.96
	Permeability (%)	83.25 ± 3.31 ^B^	91.58 ± 7.19 ^A^	85.71 ± 3.99 ^AB^	84.22 ± 3.57 ^AB^	77.85 ± 4.34 ^B^
	K^+^ (%)	\	3.40 ± 0.18 ^A^	3.46 ± 0.18 ^A^	3.27 ± 0.16 ^A^	\
	Theoretical value	\	1.9	1.9	1.9	\
	Permeability (%)	\	1.79 ± 0.010 ^A^	1.82 ± 0.10 ^A^	1.72 ± 0.09 ^A^	\
	Mg^2+^ (%)	\	0.21 ± 0.02 ^A^	0.17 ± 0.04 ^A^	\	0.17 ± 0.01 ^A^
	Theoretical value	\	0.81	0.81	\	0.81
	Permeability (%)	\	25.92 ± 0.02 ^A^	20.98 ± 0.05 ^A^	\	20.98 ± 0.01 ^A^
Physical and chemical properties	Weight loss (%)	55.68 ± 0.15 ^A^	50.25 ± 1.90 ^AB^	47.42 ± 1.34 ^B^	51.93 ± 2.39 ^AB^	45.46 ± 2.39 ^B^
	Moisture (%)	27.59 ± 3.44 ^A^	25.05 ± 1.91 ^A^	24.56 ± 0.64 ^A^	24.29 ± 2.41 ^A^	24.31 ± 1.09 ^A^
	*A*_w_	0.707 ± 0.011 ^BC^	0.723 ± 0.012 ^AB^	0.729 ± 0.007 ^A^	0.704 ± 0.024 ^C^	0.708 ± 0.006 ^ABC^
	pH	6.11 ± 0.10 ^A^	5.86 ± 0.05 ^B^	5.93 ± 0.21 ^AB^	5.95 ± 0.09 ^AB^	5.90 ± 0.13 ^B^
	Shear force (N)	402.20 ± 45.90 ^B^	338.10 ± 44.50 ^AB^	287.10 ± 46.00 ^B^	309.00 ± 31.90 ^B^	269.06 ± 16.83 ^B^
Color	L*	49.55 ± 2.32 ^A^	46.9 ± 1.19 ^AB^	45.69 ± 1.67 ^AB^	43.37 ± 4.33 ^B^	44.61 ± 4.18 ^AB^
	a*	12.21 ± 1.07 ^AB^	14.15 ± 2.41 ^A^	11.42 ± 0.76 ^B^	12.19 ± 0.37 ^AB^	11.72 ± 0.63 ^B^
	b*	26.00 ± 2.60 ^A^	24.05 ± 1.53 ^AB^	23.02 ± 0.54 ^AB^	23.67 ± 1.45 ^AB^	21.72 ± 0.90 ^B^

^A–D^ means entries in the same column without common letters are significantly different (*p* < 0.05). S: 100% NaCl; SPMA: 59.375% NaCl+28% KCl+12% MgCl_2_+0.625% L-arginine (Arg); SPM: 60% NaCl+28% KCl+12% MgCl_2_; SP: 72% NaCl+28% KCl; SM: 88% NaCl+12% MgCl_2_.

**Table 2:** Volatile compounds with OAV > 1 in dry-cured meat with different low-sodium salts.

Volatile Compounds	Threshold (μg/kg)	S	SPMA	SPM	SP	SM
3-Methylbutanal	1.2	6.04	4.83	9.42	8.28	15.23
2-Methylbutyral	0.1	67.80	52.90	98.70	113.70	180.30
Pentanal	0.85	7.41	190.25	12.04	14.11	21.89
Hexanal	5	29.54	32.34	37.76	54.43	42.70
Heptaldehyde	0.26	87.15	95.81	104.73	238.46	146.08
Octyl aldehyde	0.7	36.04	37.81	46.51	123.30	56.86
1-Nonanal	1	48.00	56.39	73.83	120.54	75.31
(*E*)-2-Heptanal	2.4	ND	2.85	3.51	ND	ND
(*E*)-2-Octenal	0.25	18.56	17.08	37.72	29.36	24.60
Decyl aldehyde	0.4	14.43	14.90	29.88	46.40	24.88
(*E*)-2-Nonenal	0.05	39.80	46.80	102.80	135.20	45.60
3-Methyl-1-butanol	1.7	ND	ND	1.89	ND	ND
1-Pentanol	0.36	168.44	128.56	60.89	72.11	68.33
1-Hexanol	10	4.43	3.77	4.58	5.01	4.82
1-Octen-3-ol	1	ND	ND	39.14	38.77	54.76
1-Heptanol	4.8	1.80	1.76	2.31	3.08	6.16
1-Octanol	2.7	ND	3.83	5.30	7.08	5.52
Methyl butyrate	15.1	0.20	0.42	0.35	0.13	1.36
Methyl 2-methylbutyrate	0.00025	36,480.00	22,720.00	26,520.00	13,400.00	72,280.00
Methyl Isovalerate	2.2	5.85	3.22	3.49	ND	9.55
Methyl pentanoate	2.2	7.72	9.48	6.26	1.35	0.00
Methyl caproate	10	8.07	12.61	7.15	1.52	14.98
Ethyl caproate	1	2.06	3.35	4.68	ND	11.69
γ-Caprolactone	0.26	38.58	27.85	0.00	55.65	53.73
γ-octa lactone	0.0222	0.00	0.00	592.34	ND	ND
2-Heptanone	0.9	8.34	10.10	8.34	13.99	18.08
6-Methyl-5-hepten-2-one	0.068	24.12	34.41	43.53	ND	59.85
2,3,5-Trimethylpyrazine	0.19	52.26	ND	27.00	91.74	44.32
Tetramethylpyrazine	0.69	17.84	ND	11.57	27.93	ND
Toluene	0.527	92.45	ND	9.92	10.53	21.76
Ethylbenzene	1.2	1.61	ND	ND	1.94	2.43
Dipentene	0.21	ND	ND	ND	19.38	48.52
2-Pentyl furan	0.27	51.74	62.37	46.15	81.15	106.81

S: 100% NaCl; SPMA: 59.375% NaCl+28% KCl+12% MgCl_2_+0.625% L-arginine (Arg); SPM: 60% NaCl+28% KCl+12% MgCl_2_; SP: 72% NaCl+28% KCl; SM: 88% NaCl+12% MgCl_2._

**Table 3:** Contents of free amino acids in dry-cured meat with the addition of different low-sodium salts.

Content (mg/100 g)	S	SPMA	SPM	SP	SM	Threshold
Asp	35.4 ± 6.5 ^C^	62.9 ± 7.1 ^B^	39.2 ± 3.5 ^C^	28.8 ± 2.9 ^C^	96.8 ± 2.7 ^A^	100
Glu	201.3 ± 24.1 ^AD^	279.1 ± 6.9 ^AB^	229.4 ± 12.2 ^CD^	302.1 ± 5.7 ^BC^	302.1 ± 0.6 ^d^	30
Ser	42.9 ± 26.2 ^A^	35.8 ± 4.1 ^A^	29.4 ± 2.0 ^A^	32.5 ± 5.3 ^A^	30.7 ± 3.8 ^A^	150
His	65.2 ± 13.0 ^A^	82.6 ± 5.8 ^A^	69.7 ± 3.1 ^A^	81.2 ± 8.9 ^A^	79.5 ± 4.5 ^A^	20
Gly	56 ± 3.2 ^C^	97.6 ± 3.9 ^AB^	77.4 ± 4.0 ^CB^	89.4 ± 5.6 ^ABC^	118.6 ± 26.5 ^A^	130
Thr	73.5 ± 4.5 ^B^	100.7 ± 1.5 ^A^	98.2 ± 5.8 ^AB^	101.6 ± 12.5 ^A^	116.7 ± 16.0 ^A^	260
Arg	94.6 ± 3.3 ^C^	137.5 ± 5.9 ^A^	96.2 ± 0.9 ^C^	91.4 ± 3.7 ^C^	125.1 ± 5.2 ^B^	50
Ala	661.8 ± 18.9 ^B^	650.7 ± 20.3 ^BC^	681.6 ± 28.5 ^B^	795.1 ± 24.8 ^A^	600.8 ± 15.0 ^C^	60
Tyr	55.5 ± 2.6 ^A^	40.5 ± 23.5 ^A^	63.8 ± 4.4 ^A^	57.8 ± 3.1 ^A^	67.1 ± 1.7 ^A^	-
Val	124.4 ± 3.5 ^A^	166.3 ± 35.0 ^A^	147.6 ± 4.4 ^A^	159.8 ± 3.2 ^A^	156.7 ± 6.9 ^A^	40
Met	40.3 ± 1.7 ^AB^	380.0 ± 8.2 ^B^	50.7 ± 2.0 ^A^	37.6 ± 2.7 ^B^	46.5 ± 1.8 ^AB^	30
Phe	54.7 ± 1.3 ^A^	80.2 ± 29.1 ^A^	70.2 ± 3.1 ^A^	52.4 ± 1.4 ^A^	65.3 ± 0.3 ^A^	90
Ile	64.4 ± 1.0 ^A^	82.0 ± 17.2 ^A^	83.4 ± 2.5 ^A^	69.7 ± 3.3 ^A^	78.6 ± 2.1 ^A^	90
Leu	94.8 ± 4.4 ^D^	111.2 ± 5.5 ^CD^	128.6 ± 6.3 ^A^	99.3 ± 3.8 ^CD^	117.7 ± 0.6 ^AB^	190
Lys	175.4 ± 2.4 ^D^	226.3 ± 9.8 ^B^	208.7 ± 7.9 ^BC^	205.2 ± 3.9 ^C^	257.6 ± 8.3 ^A^	50
Pro	94.0 ± 21.5 ^A^	101.7 ± 27.4 ^A^	83.2 ± 44.0 ^A^	110.1 ± 14.2 ^A^	127.3 ± 21.2 ^A^	300
Cys	4.1 ± 3.0 ^A^	5.1 ± 5.0 ^A^	2.9 ± 1.5 ^A^	238.6 ± 4.3 ^A^	4.7 ± 3.8 ^A^	
Umami FAAs	236.8 ± 30.3 ^D^	342.0 ± 13.7 ^B^	268.6 ± 9.4 ^CD^	283.65 ± 0.4 ^C^	398.8 ± 6.7 ^A^	-
Sweet FAAs	834.1 ± 8.7 ^B^	884.8 ± 27.2 ^B^	886.6 ± 39.5 ^B^	1018.6 ± 47.0 ^A^	866.8 ± 34.1 ^B^	-
Bitter FAAs	334.6 ± 21.5 ^C^	396.5 ± 22.4 ^AB^	415.6 ± 18.3 ^A^	360.4 ± 12.4 ^BC^	408.1 ± 7.2 ^A^	-
Total FAAs	1938.3 ± 46.0 ^C^	2298.3 ± 49.0 ^AB^	2160.0 ± 86.6 ^B^	2269.2 ± 78.1 ^AB^	2391.7 ± 2.0 ^A^	-

Umami amino acids include aspartic acid (Asp) and glutamic acid (Glu); sweet amino acids include alanine (Ala), glycine (Gly), serine (Ser), and threonine (Thr); bitter amino acids include histidine (His), phenylalanine (Phe), tyrosine (Tyr), and leucine (Leu). The threshold unit is mg/100g. ^A–D^ mean values in the same row not followed by a common letter differ significantly (*p* < 0.05). S: 100% NaCl; SPMA: 59.375% NaCl+28% KCl+12% MgCl_2_+0.625% L-arginine (Arg); SPM: 60% NaCl+28% KCl+12% MgCl_2_; SP: 72% NaCl+28% KCl; SM: 88% NaCl+12% MgCl_2_.

**Table 4:** Sensory evaluation scores of dry-cured meat with different low-sodium salt formulations.

	S	SPMA	SPM	SP	SM
	Score				
Redness	2.53 ± 0.99 ^A^	3.08 ± 1.14 ^A^	2.67 ± 1.23 ^A^	3.17 ± 1.10 ^A^	3.15 ± 1.32 ^A^
Homogeneity	3.11 ± 0.96 ^A^	3.33 ± 1.08 ^A^	3.17 ± 1.10 ^A^	3.00 ± 1.14 ^A^	3.22 ± 1.22 ^A^
Gloss	2.73 ± 0.91 ^A^	3.18 ± 0.87 ^A^	3.03 ± 1.12 ^A^	2.61 ± 0.78 ^A^	3.25 ± 0.97 ^A^
Saltiness	3.83 ± 1.14 ^A^	3.99 ± 0.76 ^A^	3.44 ± 0.78 ^A^	3.88 ± 0.95 ^A^	4.02 ± 0.84 ^A^
Bitterness	1.42 ± 0.77 ^A^	1.69 ± 0.89 ^A^	1.86 ± 1.16 ^A^	1.81 ± 0.93 ^A^	1.53 ± 0.81 ^A^
Umami	2.12 ± 1.09 ^A^	2.56 ± 0.99 ^A^	2.39 ± 1.05 ^A^	2.33 ± 1.08 ^A^	2.62 ± 1.10 ^A^
Sweetness	1.36 ± 0.72 ^A^	1.31 ± 0.62 ^A^	1.36 ± 0.64 ^A^	1.36 ± 0.64 ^A^	1.42 ± 0.65 ^A^
Hardness	2.93 ± 0.93 ^A^	3.00 ± 0.69 ^A^	2.96 ± 1.12 ^A^	3.32 ± 1.01 ^A^	2.99 ± 0.83 ^A^
Fat adhesion	2.33 ± 1.03 ^A^	2.44 ± 1.10 ^A^	2.22 ± 0.94 ^A^	2.28 ± 1.02 ^A^	2.72 ± 1.07 ^A^
Chewiness	3.03 ± 0.96 ^A^	3.47 ± 0.88 ^A^	3.08 ± 1.03 ^A^	3.12 ± 0.77 ^A^	3.36 ± 1.11 ^A^
Acceptance	3.31 ± 1.05 ^A^	3.08 ± 1.29 ^A^	3.04 ± 1.24 ^A^	3.06 ± 0.87 ^A^	3.08 ± 1.09 ^A^

S: 100% NaCl; SPMA: 59.375% NaCl+28% KCl+12% MgCl_2_+0.625% L-arginine (Arg); SPM: 60% NaCl+28% KCl+12% MgCl_2_; SP: 72% NaCl+28% KCl; SM: 88% NaCl+12% MgCl_2_. Different letters in the same group indicate significant differences (*p* < 0.05; Tukey test).

**Table 5:** Sensory attributes and assessment criteria for sensory evaluation.

Category	Sensory Attributes	Judgment Criteria (a Scale of 1 to 5)
Appearance	Redness	Pale pink to dark red
	Homogeneity	Non-homogeneous to homogeneous
	Glossiness	Glossy from dark to bright
Taste	Saltiness	Not distinctly salty to moderately salty
	Bitterness	Not bitter to distinctly bitter
	Umami	Not perceived to perceived
	Sweetness	Not very sweet to very sweet
Texture	Meat hardness	Loose to very hard in texture
	Fat adhesiveness	Not adhesive to appropriately adhesive
	Chewiness	Poorly chewable to very chewable
Overall acceptance	Overall acceptance	Unattractive to very attractive

## Data Availability

The original contributions presented in the study are included in the article/Supplementary Material.

## Supplementary Materials

The following supporting information can be downloaded at: https://www.mdpi.com/article/10.3390/molecules29102194/s1, Table S1: Content of volatile compounds in dry-cured meat with the addition of different low-sodium salts.
