# Supplementary material for: Quality and Flavor Difference in Dry-Cured Meat Treated with Low-Sodium Salts: An Emphasis on Magnesium
Source: Molecules. 2024 May 8;29(10):2194. doi: 10.3390/molecules29102194 (PMC11124267; doi:10.3390/molecules29102194)
Supplement: Supplementary file 1 [file molecules-29-02194-s001.zip › molecules-2929946-supplementary.pdf]

Supplementary Table S1. Content of volatile compounds in dry-cured meat with the addition of different low-sodium salts.

	RI	Volatile compounds	Content (μg/kg)				
			S	SPMA	SPM	SP	SM
**Aldehydes**							
1	913	3-Methylbutanal	$7.25±1.56^{AB}$	$5.80±0.53^{B}$	$11.30±2.59^{AB}$	$9.94±1.74^{AB}$	$18.27±2.71^{A}$
2	933	2-Methylbutyral	$6.78±1.49^{AB}$	$5.29±0.27^{B}$	$9.87±1.96^{AB}$	$11.37±1.12^{AB}$	$18.03±2.94^{A}$
3	974	Pentanal	$6.30±0.14^{B}$	$7.60±0.23^{AB}$	$10.23±2.07^{AB}$	$11.99±2.32^{AB}$	$18.61±2.81^{A}$
4	1080	Hexanal	$147.72±38.75^{A}$	$161.71±32.07^{A}$	$188.78±23.33^{A}$	$272.14±98.49^{A}$	$213.52±1.87^{A}$
5	1182	Heptaldehyde	$22.66±4.19^{B}$	$24.91±0.02^{B}$	$27.23±3.57^{B}$	$62.00±10.54^{A}$	$37.98±3.13^{AB}$
6	1278	Octyl aldehyde	$25.23±1.99^{B}$	$26.47±1.33^{B}$	$32.56±1.90^{B}$	$86.31±11.62^{A}$	$39.80±2.01^{B}$
7	1380	1-Nonanal	$48.00±2.43^{B}$	$56.39±0.59^{B}$	$73.83±6.53^{B}$	$120.54±12.08^{A}$	$75.31±2.22^{B}$
8	1315	(E)-2-Heptanal	ND	$6.84±0.79^{A}$	$8.43±0.80^{A}$	ND	ND
9	1424	(E)-2-Octenal	$4.64±1.10^{A}$	$4.27±2.63^{A}$	$9.43±0.94^{A}$	$7.34±0.64^{A}$	$6.15±2.28^{A}$
10	1495	Decyl aldehyde	$5.77±1.58^{B}$	$5.96±0.23^{B}$	$11.95±0.07^{AB}$	$18.56±3.17^{A}$	$9.95±0.65^{AB}$
11	1508	Benzaldehyde	$6.06±0.85^{A}$	$5.42±0.35^{A}$	$7.72±0.18^{A}$	$10.75±0.77^{A}$	$9.25±3.01^{A}$
12	1529	(E)-2-Nonenal	$1.99±0.19^{B}$	$2.34±0.10^{B}$	$5.14±0.24^{A}$	$6.76±1.28^{A}$	$2.28±0.16^{B}$
13	2133	Hexadecanal	ND	ND	ND	ND	4.28±0.47
		Total	$282.41±50.16^{B}$	$311.81±25.81^{B}$	$396.46±54.57^{AB}$	$617.68±143.78^{A}$	$457.40±20.30^{AB}$
**Alcohols**							
14	1161	1-Penten-3-ol	ND	ND	ND	ND	2.99±0.38
15	1185	3-Methyl-1-butanol	ND	ND	$3.22±0.27^{A}$	ND	$6.02±2.62^{A}$
16	1260	1-Pentanol	$21.83±2.38^{A}$	$16.66±0.12^{A}$	$21.92±3.33^{A}$	$25.96±7.48^{A}$	$24.60±2.94^{A}$
17	1362	1-Hexanol	$44.26±7.55^{A}$	$37.71±1.48^{A}$	$45.75±1.13^{A}$	$50.14±6.44^{A}$	$48.18±4.77^{A}$
18	1430	1-Octen-3-ol	ND	ND	$39.14±2.22^{A}$	$38.77±1.98^{A}$	$54.76±7.36^{A}$

19	1447	1-Heptanol	8.64±1.13^{B}	8.47±0.03^{B}	11.11±0.11^{B}	14.76±0.19^{B}	29.58±2.85^{A}
20	1542	2,3-Butanediol	13.06±0.24^{B}	20.38±0.51^{AB}	23.19±1.93^{A}	18.11±0.61^{AB}	26.33±1.60^{A}
21	1567	1-octanol	ND	10.33±0.43^{B}	14.30±0.55^{AB}	19.12±0.74^{A}	14.90±1.64^{AB}
		Total	87.80±13.92^{B}	93.55±2.59^{B}	158.64±19.11^{A}	166.82±15.84^{A}	198.36±5.07^{A}
**Esters**							
22	910	Methyl isobutyrate	ND	ND	ND	ND	3.09±0.43
23	976	Methyl butyrate	2.95±0.58^{B}	6.39±1.19^{B}	5.32±1.41^{B}	1.89±0.33^{B}	20.66±3.80^{A}
24	1010	Methyl 2-methylbutyrate	9.12±3.24^{AB}	5.68±0.58^{AB}	6.63±2.14^{AB}	3.35±0.61^{B}	18.07±3.32^{A}
25	1018	Methyl Isovalerate	12.88±5.98^{A}	7.08±1.11^{A}	7.68±2.66^{A}	ND	21.02±3.63^{A}
26	1089	Methyl pentanoate	16.98±6.99^{AB}	20.85±3.09^{A}	13.78±2.25^{AB}	2.97±0.02^{B}	ND
27	1177	Methyl caproate	80.69±39.51^{AB}	126.10±0.38^{AB}	71.48±15.58^{AB}	15.20±3.55^{B}	149.75±27.24^{A}
28	1223	Ethyl caproate	2.06±0.52^{B}	3.35±0.16^{B}	4.68±0.21^{B}	ND	11.69±1.27^{A}
29	1268	Hexyl acetate	0.76±0.13	ND	ND	ND	ND
30	1429	Ethyl caprylate	ND	ND	ND	ND	3.99±0.94
31	1678	γ-caprolactone	10.03±0.55^{A}	7.24±3.88^{A}	ND	14.47±0.60^{A}	13.97±1.22^{A}
32	1906	γ-octalactone	ND	ND	13.15±0.13	ND	ND
33	2028	γ-nonanoic lactone	ND	2.31±0.14	ND	ND	ND
		Total	135.47±54.43^{AB}	178.99±10.26^{AB}	122.73±24.18^{AB}	37.89±5.10^{B}	242.86±83.15^{A}
**Ketones**							
34	811	Acetone	ND	1.85±0.07^{A}	ND	4.82±0.52^{A}	ND
35	1068	2,3-Pentane-dione	ND	1.64±0.60	ND	ND	ND
36	1183	2-Heptanone	7.51±2.91^{C}	9.09±0.18^{AB}	7.51±0.41^{C}	12.59±1.88^{AB}	16.27±1.22^{A}
37	1277	3-Hydroxybutan-2-one	ND	5.39±0.31^{B}	15.33±1.06^{AB}	11.81±0.76^{B}	22.96±2.22^{A}
38	1296	2-Octanone	3.53±0.76^{A}	4.93±0.26^{A}	3.84±0.15^{A}	5.08±0.65^{A}	ND
39	1320	2-methyloctan-3-one	ND	9.43±0.74^{A}	7.67±0.36^{A}	7.94±0.39^{A}	ND
40	1325	6-Methyl-5-hepten-2-one	1.64±0.07^{A}	2.34±0.19^{A}	2.96±0.16^{A}	ND	4.07±0.65^{A}

41	1382	2-Nonanone	ND	4.17±0.35	ND	ND	ND
		Total	12.69±4.57^{B}	38.85±1.35^{A}	37.33±3.90^{A}	42.24±4.20^{A}	43.30±8.18^{A}
**Alkanes**							
42	797	2,4-Dimethylheptane	4.36±2.31^{B}	11.82±1.12^{B}	5.56±0.33^{B}	12.63±2.40^{B}	39.10±4.88^{A}
43	1296	Tridecane	ND	13.24±0.05	ND	ND	ND
44	1483	2-Methyltetradecane	2.40±0.12^{A}	ND	2.95±0.46^{A}	ND	ND
45	1154	1-Chloroheptane	ND	ND	ND	2.39±0.60^{A}	2.24±0.01^{A}
		Total	6.76±2.42^{B}	25.07±2.28^{AB}	8.51±1.24^{B}	15.03±3.01^{B}	41.33±9.79^{A}
**Pyrazines**							
46	1401	2,3,5-Trimethylpyrazine	9.93±3.14^{AB}	ND	5.13±0.10^{B}	17.43±1.55^{A}	8.42±1.02^{B}
47	1466	Tetramethylpyrazine	12.31±2.36^{B}	ND	7.98±0.07^{B}	19.27±0.58^{A}	ND
		Total	22.34±5.35^{B}	ND	13.11±0.33^{BC}	36.70±2.14^{A}	8.42±2.04^{C}
**Aromatic**							
48	1035	Toluene	65.93±37.19^{A}	ND	5.23±0.96^{A}	5.55±1.17^{A}	11.47±2.51^{A}
49	1122	Ethylbenzene	1.93±0.62^{A}	ND	ND	2.33±1.08^{A}	2.91±0.19^{A}
50	1128	M-xylene	2.27±0.10^{A}	2.35±0.44^{A}	2.90±2.16^{A}	4.65±1.81^{A}	6.67±0.94^{A}
51	1175	O-xylene	ND	1.14±0.22^{A}	ND	2.45±0.50^{A}	2.91±0.24^{A}
		Total	70.14±36.67^{A}	3.50±0.66^{B}	8.14±3.12^{AB}	14.98±4.55^{AB}	23.96±2.26^{AB}
**Olefins**							
52	833	1-Octene	ND	ND	0.83±0.06^{A}	1.43±0.44^{A}	1.81±0.20^{A}
53	864	trans-2-Octene	ND	1.51±0.32^{A}	ND	ND	4.51±0.58^{A}
54	1180	Dipentene	ND	ND	ND	4.07±0.30^{A}	10.19±1.60^{A}
		Total	ND	1.51±0.33^{B}	0.84±0.06^{B}	5.50±0.74^{B}	16.51±4.76^{A}
**Furans**							
55	1230	2-Pentyl furan	13.97±8.51^{A}	16.84±0.13^{A}	12.46±0.34^{A}	21.91±2.27^{A}	28.84±3.45^{A}
		Total	13.97±8.51^{A}	16.84±0.13^{A}	12.46±0.34^{A}	21.91±2.27^{A}	28.84±3.45^{A}

The different letters in the same raw represent significant differences in the results of the Tukey test ($p < 0.05$). RI—retention indices calculated concerning the retention time of n-alkane (C5–C25); ND — not detected. S: 100% NaCl; SPMA: 59.375% NaCl+28% KCl+12% $MgCl_2$+0.625% L-arginine (Arg); SPM: 60% NaCl+28% KCl+12% $MgCl_2$; SP: 72% NaCl+28% KCl; SM: 88% NaCl+12% $MgCl_2$
